# Sustainable Flame-Retardant PLA Composites Incorporating Raw Wood-Derived Biochar and Magnesium Hydroxide

**DOI:** 10.3390/ma19132792

**Published:** 2026-07-01

**Authors:** Yuxin Liu, Jinfeng Zhang, António Benjamim Mapossa, Maryam Rasouli, Uttandaraman Sundararaj

**Affiliations:** Department of Chemical and Petroleum Engineering, University of Calgary, Calgary, AB T2N 1N4, Canada; jinfeng.zhang1@ucalgary.ca (J.Z.); mapossabenjox@gmail.com (A.B.M.); maryam.rasouli@ucalgary.ca (M.R.)

**Keywords:** PLA, biochar, magnesium hydroxide (MH), flame retardant, sustainable composites

## Abstract

The development of sustainable flame-retardant polymer composites is important for expanding the practical use of bio-based plastics while reducing reliance on petroleum-derived and halogenated materials. In this work, biodegradable polylactic acid (PLA) composites were prepared using raw wood-derived biochar as a degradable carbon-based filler and magnesium hydroxide (MH) as a halogen-free flame-retardant additive. PLA/Biochar/MH composites were prepared by melt compounding and compression molding, followed by systematic evaluation of their structural, thermal, flame-retardant, mechanical, and stability-related properties. The flame-retardant performance, evaluated by limiting oxygen index (LOI) and UL-94 (UL: Underwriters Laboratories) vertical burning tests, was significantly enhanced by the combined biochar/MH system. Biochar alone slightly increased the LOI of PLA, while MH-containing composites exceeded the practical 21% LOI threshold, with PLA/Biochar20/MH20 achieving the highest LOI value of 26.2%. This improvement was attributed to char formation, heat absorption, gas dilution, and magnesium oxide-supported barrier formation. The composites also maintained reasonable dimensional stability after accelerated aging with thickness changes below 1%. Overall, this study demonstrates that combining biodegradable PLA with degradable biochar and halogen-free MH provides a promising sustainable strategy for developing flame-retardant PLA-based composites with improved residue formation and dimensional stability.

## 1. Introduction

The growing demand for sustainable materials has accelerated the development of bio-based polymers as alternatives to petroleum-derived plastics [1,2]. Among them, polylactic acid (PLA) has become one of the most widely studied biodegradable polyesters due to its renewable origin, commercial availability, good processability, high stiffness, and relatively high mechanical strength [3,4]. These characteristics make PLA attractive for packaging, consumer products, biomedical materials, and emerging durable applications [5]. However, despite these advantages, the broader use of PLA remains limited by its inherent brittleness, limited impact resistance [6], relatively low heat resistance, and flammability [7,8]. Therefore, developing PLA-based materials with improved functional performance while maintaining their sustainable character is an important research direction.

Biochar has recently gained increasing attention as a degradable carbon-based filler for polymer composites [9]. It is typically produced through the thermochemical conversion of renewable biomass or organic waste, offering a value-added pathway for waste utilization and carbon-rich material production [10,11,12]. Compared with conventional mineral fillers, biochar is attractive because of its low cost [13], tunable surface chemistry [14], low density [15], high thermal stability [16], and carbonaceous structure [17]. These features allow biochar to function not only as a reinforcing filler but also as a functional additive capable of influencing thermal degradation, char formation, and dimensional stability in polymer matrices. For example, Alghyamah et al. [18] incorporated biochar into polypropylene (PP) composites and reported that biochar improved the thermal stability of PP, with the degradation temperature increasing by up to 80 °C compared with neat PP, demonstrating the effectiveness of biochar as a sustainable functional filler in polymer matrices.

The incorporation of biochar into PLA provides a promising strategy for improving the performance and sustainability of PLA-based composites. The rigid carbonaceous structure of biochar can enhance stiffness and thermal resistance [16], while its char-forming ability may contribute to improved flame-retardant behavior by promoting condensed-phase protection during combustion [19]. In addition, using biochar as a filler can partially replace polymer content, reduce material cost, and increase the bio-based or waste-derived fraction of the final composite [9,20]. These advantages align well with the development of circular and low-carbon polymer materials. However, the performance of PLA/biochar composites strongly depends on filler loading, dispersion quality, interfacial compatibility, and processing conditions [21]. While biochar can improve stiffness and thermal stability, excessive loading or poor interfacial adhesion may introduce stress-concentration sites, leading to reduced toughness and impact resistance. This issue is particularly important for PLA, which is already brittle compared with many conventional polymers [21,22]. Therefore, achieving a balance between improved thermal/flame-related performance and retained mechanical toughness remains a key challenge in the design of PLA/biochar composites.

Recent studies have emphasized the importance of renewable, bio-derived, and mineral-based additives for improving the flame resistance of polymeric materials. Chen et al. [23] reviewed recent advances in bio-derived flame retardants and highlighted that renewable additives can promote char formation and improve flame-retardant behavior in polymer matrices. In addition, silica-based particles have been reported to enhance the flame-retardant properties of polyurethane aerogels [24], with the best performance observed at 2 wt.% SiO_2_ loading and up to an eightfold increase in flame retardancy, further demonstrating the potential of inorganic or mineral-rich additives in improving fire resistance. However, although biochar has been studied as a sustainable filler in polymer composites, its combined use with magnesium hydroxide in PLA has not been sufficiently investigated, particularly with respect to balancing flame resistance, residue formation, impact strength, water absorption, and dimensional stability.

In this study, PLA/biochar composites were developed through melt compounding followed by compression molding, with magnesium hydroxide (MH) incorporated as a halogen-free flame-retardant additive. The effects of biochar and MH on morphology, chemical structure, thermal degradation behavior, limiting oxygen index (LOI), UL-94 vertical burning behavior, impact strength, water absorption, and dimensional stability were systematically investigated. Particular attention was given to the use of biochar as a sustainable carbon-based filler together with halogen-free MH in enhancing the flame resistance and residue formation of PLA composites while evaluating the associated trade-offs in mechanical and stability-related properties. This work therefore provides insight into the potential of biochar/MH systems for developing more sustainable flame-retardant PLA-based composites.

## 2. Experiment

### 2.1. Materials

Polylactic acid (PLA) pellets (NatureWorks^®^ Ingeo™ 4032D) with a melt flow index (MFI) of 7 g/10 min (2.16 kg at 210 °C) were obtained from NatureWorks (Blair, NE, USA). Commercial biochar was received from Canadian Agrichar Inc. (Maple Ridge, BC, Canada). According to the supplier, the biochar was produced by thermochemical carbonization/pyrolysis of biomass under oxygen-limited conditions at temperatures below 700 °C. Detailed production parameters, including exact feedstock composition, pyrolysis temperature, residence time, and heating rate, were not available. MH was purchased from Sigma-Aldrich (St. Louis, MO, USA). All chemicals were used without further purification.

### 2.2. Preparation of Composites

PLA/Biochar/MH composites were prepared using a Haake torque rheometer equipped with a drum-type mixing blade. The formulations contained biochar at 20 or 30 wt.% and magnesium hydroxide (MH) at 10 or 20 wt.%, as summarized in Table 1. Before compounding, PLA, biochar, and MH were dried overnight in a vacuum oven at 60 °C to remove residual moisture. The dried components were then added simultaneously into the Haake torque rheometer (Thermo Fisher Scientific, Waltham, MA, USA) and melt-mixed at 180 °C and 90 rpm for 8 min. After mixing, the obtained composites were granulated and subsequently compression-molded using a Carver hot press (Carver Inc., Wabash, IN, USA) to produce rectangular specimens. Compression molding was carried out at 180 °C for 8 min under a pressure of 40 MPa. The molded samples were then used for further characterization, including limiting oxygen index, impact testing, water absorption, and dimensional stability measurements. The neat PLA pellets were white in appearance, whereas the PLA/biochar composites became black after biochar incorporation due to the carbonaceous nature of biochar.

### 2.3. Characterizations

The morphology of the composites was examined by scanning electron microscopy (SEM) using a high-resolution Philips XL30 microscope (FEI, Hillsboro, OR, USA). Fourier transform infrared spectroscopy (FTIR) was conducted using a Cary 630 FTIR spectrometer (Agilent Technologies, Santa Clara, CA, USA) to identify the functional groups present in the composites. Each spectrum was collected over 16 scans. The thermal degradation behavior of the samples was evaluated by thermogravimetric analysis (TGA) using a TGA Q550 instrument (TA Instruments, New Castle, DE, USA). The samples were heated from room temperature to 900 °C at a heating rate of 10 °C/min under a nitrogen atmosphere with a flow rate of 50 mL/min.

The notched Izod impact strength of the composites was measured at room temperature using a pendulum impact tester in accordance with ASTM D256-24 [25]. V-notched specimens were prepared using a Qualitest QC-640A impact specimen notch cutter (Qualitest, Lauderdale, FL, USA). For each formulation, five specimens were tested, and the average impact strength was reported in kJ/m^2^.

The flame-retardant performance of the composites was assessed using the limiting oxygen index (LOI) test, which measures the minimum oxygen concentration in an oxygen/nitrogen mixture required to sustain flaming combustion. LOI testing was carried out using an AT-P6012A instrument (Amade Technology Co., Limited, Hong Kong, China) following ASTM D2863-19 [26]. During the test, the specimen bars were mounted vertically and ignited with a burner. After 20 s of ignition, the flame source was removed. If the specimen self-extinguished before burning for 3 min or before the flame front traveled 5 cm, the oxygen concentration gradually increased until the limiting oxygen concentration was determined. UL-94 vertical flammability tests were performed following ASTM D3801-20A [27], using specimens with dimensions of 127 mm × 12.7 mm × 4.0 mm.

Water absorption behavior was evaluated in accordance with ASTM D570-22 [28]. The preconditioned specimens were immersed in distilled water at 25 °C. After immersion periods of 48 h, the samples were removed, gently wiped with a dry cloth to eliminate surface water, and immediately weighed to the nearest 0.001 g. Three specimens were tested for each formulation, and the water uptake was determined from the change in sample mass using Equation (1) [29].

(1) $$W(\%)=\frac{\left(m_{1}-m_{0}\right)}{m_{0}}\times 100$$

where W represents the water absorption percentage, while $m_{0}$ and $m_{1}$ denote the sample mass before and after immersion, respectively. For each composite formulation, three specimens were tested, and the standard deviation was calculated from the measured water absorption values.

Accelerated thermal aging was conducted to evaluate the dimensional stability of the composites under simulated service conditions. The specimens were aged at 60 °C in a temperature-controlled oven. After 48 h, the samples were removed and conditioned at 25 °C for 4 h before measurement. Thickness changes were measured at 10 different points using a digital vernier caliper, and the average expansion was recorded. The dimensional change degree, C, was calculated using Equation (2) [30].

(2) $$C\;(\%)=\frac{\left(d_{1}-d_{0}\right)}{d_{0}}\times 100$$

where $d_{0}$ and $d_{1}$ represent the specimen thickness before and after thermal aging, respectively. Three specimens were tested for each composite formulation.

For tests with replicate measurements, data are reported as mean ± standard deviation. Impact strength values were calculated from five specimens, while water absorption and dimensional change values were calculated from three specimens for each formulation. The error bars in the corresponding figures represent standard deviations. LOI values were determined according to ASTM D2863-19 [26] and are reported without error bars. UL-94 results are reported as classification outcomes according to ASTM D3801-20A [27].

## 3. Results and Discussion

### 3.1. Scanning Electron Microscopy (SEM)

**Figure 1:**
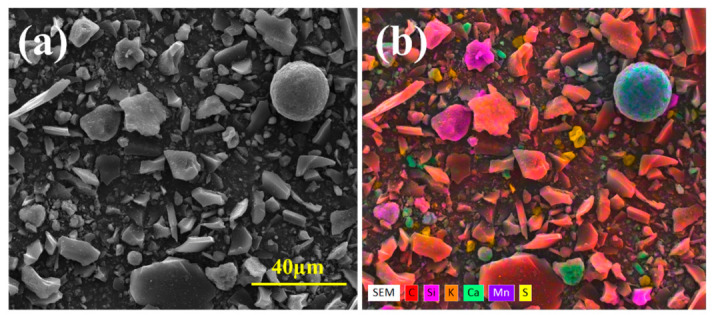
(**a**) SEM micrograph of biochar and (**b**) EDX selective elemental mapping of biochar.

**Figure 2:**
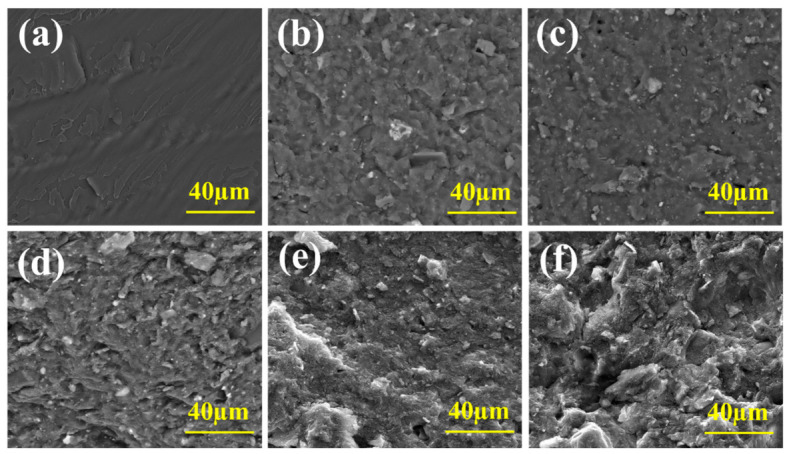
SEM micrographs of (**a**) PLA; (**b**) PLA/Biochar20; (**c**) PLA/Biochar30; (**d**) PLA/Biochar20/MH10; (**e**) PLA/Biochar30/MH10 and (**f**) PLA/Biochar20/MH20 composite.

The SEM images first reveal the characteristic morphology of raw biochar (Figure 1a), which consists of irregular, angular, and rough carbonaceous particles with a relatively porous surface. SEM-EDX elemental mapping of raw biochar is shown in Figure 1b, providing qualitative information on the elemental distribution within the biochar structure. These features are relevant to its role as a carbon-based filler, since the rough particle morphology may influence dispersion, interfacial contact with PLA, and residue formation during thermal degradation or combustion. After incorporation into PLA, the biochar particles are distributed within the polymer matrix, although localized agglomeration is still observed. Compared with neat PLA, which exhibits a relatively smooth and continuous fracture surface typical of brittle failure (Figure 2a), the PLA/Biochar and PLA/Biochar/MH composites show rougher and more heterogeneous fracture morphologies.

As shown in Figure 2b, the addition of 20 wt.% biochar results in a rougher fracture surface for PLA/Biochar20, with small biochar particles dispersed throughout the PLA matrix. When the biochar content is increased to 30 wt.% (Figure 2c), more voids and irregular features are observed, suggesting enhanced filler-induced crack deflection and increased morphological heterogeneity. With the introduction of MH, PLA/Biochar20/MH10 displays a more fractured surface with visible cavities and exposed filler particles (Figure 2d), indicating that MH further increases surface tortuosity and affects crack propagation. The presence of MH may also slightly influence the PLA matrix during melt processing, leading to more visible microcracks and localized defects. This effect becomes more pronounced at higher filler loading, as observed for PLA/Biochar30/MH10 (Figure 2e). For PLA/Biochar20/MH20 (Figure 2f), the fracture surface is the roughest and most heterogeneous, with larger exposed particles, agglomerates, and voids. This suggests that excessive filler loading may reduce dispersion quality and generate stress-concentration sites within the composite.

### 3.2. Fourier-Transform Infrared (FTIR) Spectroscopy

**Figure 3:**
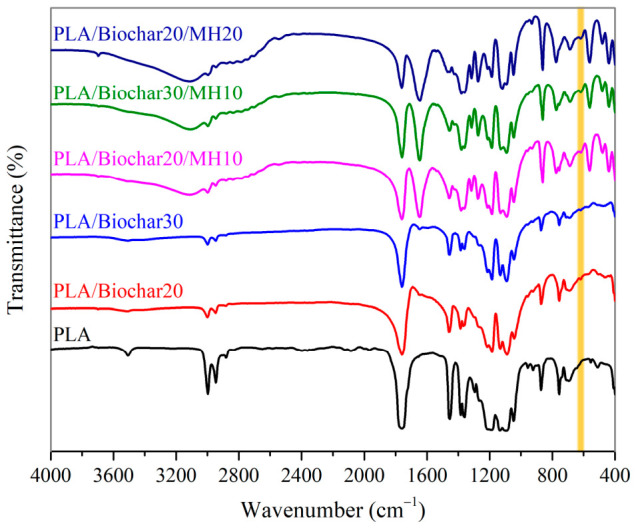
FTIR spectra of PLA composites.

The chemical structures of PLA and its composites were investigated by FTIR spectroscopy. Neat PLA shows its characteristic absorption bands, including a strong peak at approximately 1758 cm^−1^, corresponding to ester carbonyl C=O stretching, and bands at 2997 and 2946 cm^−1^, assigned to asymmetric and symmetric C–H stretching of the methyl groups. The typical ester C–O–C/C–O stretching vibrations of PLA are observed in the region of 1184–1080 cm^−1^ [31], confirming the polyester structure of the PLA matrix (Figure 3).

After the incorporation of biochar, the main characteristic peaks of PLA remain visible. However, a weak absorption band around 618 cm^−1^ appears after biochar addition, which may arise from mineral residues or aromatic vibrations associated with the carbonaceous biochar structure. For the MH-containing composites, including PLA/Biochar20/MH10, PLA/Biochar30/MH10, and PLA/Biochar20/MH20, the broad band at 3120 cm^−1^ becomes more pronounced. This broadening can be attributed to hydroxyl groups associated with MH and adsorbed moisture. It may also suggest that the presence of MH slightly affects the PLA matrix during melt processing, possibly promoting limited hydrolytic or thermal degradation [32]. This is consistent with the SEM observation that MH-containing composites exhibit rougher fracture surfaces with more microcracks and localized defects. Overall, the FTIR spectra confirm the successful incorporation of biochar and MH into PLA while preserving the main chemical features of the PLA matrix.

### 3.3. Thermogravimetric Analysis (TGA)

**Figure 4:**
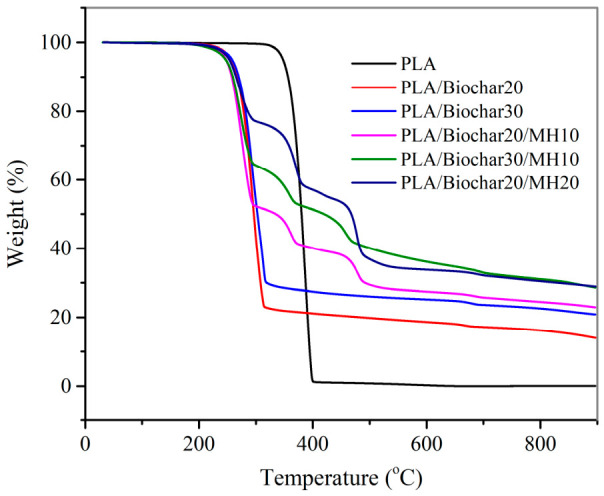
TGA thermogram graph of PLA composites.

The TGA thermograms of PLA and its composites are shown in Figure 4. Neat PLA shows the highest decomposition temperature at 320 °C, almost 100 °C higher than that of the composite samples, indicating that its resistance to thermal decomposition is higher than that of the composite samples. The onset degradation temperature of composite materials is affected by the interfacial bonding between the polymer matrix and the added filler. In this case, oxygen-containing groups on biochar may influence the interaction between biochar and the PLA matrix, thereby improving interfacial adhesion and modifying the thermal stability of the composites. Although direct evidence of specific interfacial bonding was not obtained in this study, the observed behavior is consistent with trends reported in the literature [21,33,34].

Although neat PLA shows the highest decomposition temperature, the composites retain substantially higher residual mass after thermal degradation. From Figure 4, neat PLA exhibits a sharp single-step degradation behavior, with almost complete mass loss and only 0.06% residue remaining at 900 °C. This indicates that PLA undergoes nearly complete thermal decomposition under nitrogen. After biochar incorporation, the residual weight increases markedly to 14.01% for PLA/Biochar20 and 20.86% for PLA/Biochar30, confirming the contribution of the carbonaceous biochar phase to char formation.

For the MH-containing composites, the degradation curves show more distinct multi-step weight-loss behavior. This can be explained by the overlapping PLA degradation, MH dehydration, and possible MH-assisted PLA chain scission [35]. PLA mainly decomposes through ester bond scission and volatilization, while MH decomposes endothermically to form MgO and release water vapor, typically beginning around 300–330 °C [36]. The release of water can promote hydrolytic scission of PLA [37], and the formation of MgO introduces additional mass-loss steps and leaves more inorganic residue, which explains the higher final residues of 22.91%, 28.59%, and 28.86% for PLA/Biochar20/MH10, PLA/Biochar30/MH10, and PLA/Biochar20/MH20, respectively. The MgO residue, together with biochar-derived char, can form a more stable protective layer during thermal decomposition. This behavior is consistent with the known flame-retardant mechanism of MH, which absorbs heat during decomposition, releases water vapor, and forms MgO as a protective inorganic residue. Therefore, the TGA results indicate two separate effects: neat PLA has greater resistance to the main stage of thermal decomposition, whereas the biochar- and MH-containing composites show enhanced residue formation after degradation. The increased residual mass should not be interpreted as direct evidence of improved thermal stability, but rather as evidence of increased carbonaceous/inorganic residue formation. This residue originates from the biochar phase and, in the MH-containing composites, from MgO formed during MH decomposition.

### 3.4. Flammability Properties

**Figure 5:**
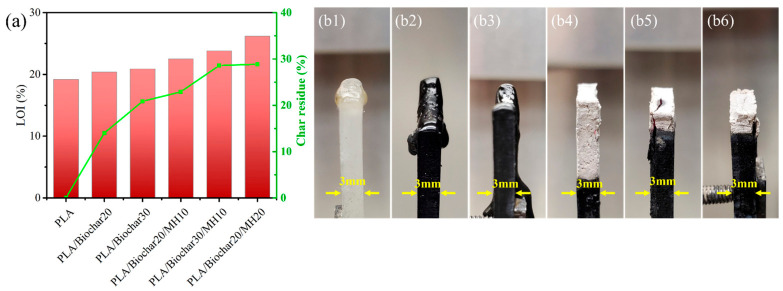
(**a**) LOI values and solid char residue of PLA composites and digital micrographs of chars formed after LOI test ((**b1**), PLA, (**b2**), PLA/Biochar20, (**b3**), PLA/Biochar30, (**b4**), PLA/Biochar20/MH10, (**b5**), PLA/Biochar30/MH10, and (**b6**), PLA/Biochar20/MH20).

The flame-retardant performance of PLA and its composites was evaluated by LOI, and the results are summarized together with the TGA residue values in Figure 5a. Neat PLA shows a relatively low LOI value of 19.2%, confirming its inherent flammability and limited ability to sustain combustion resistance. Similar findings have been reported in previous studies [38,39]. The incorporation of biochar slightly increases the LOI to 20.4% for PLA/Biochar20 and 20.9% for PLA/Biochar30. This improvement can be attributed to the carbonaceous structure of biochar, which promotes char formation and acts as a physical barrier during combustion (Figure 5(b2,b3)). The barrier layer can slow heat transfer and reduce the release of volatile degradation products from the PLA matrix.

A more pronounced improvement is observed after introducing MH into the PLA/biochar system. LOI increases to 22.5% for PLA/Biochar20/MH10 and 23.8% for PLA/Biochar30/MH10, indicating that MH works effectively with biochar to improve flame resistance. During heating or combustion, MH decomposes endothermically to release water vapor and form MgO. The released water vapor absorbs heat, suppresses smoke, and dilutes combustible gases, while the MgO residue contributes to the formation of a protective inorganic barrier, as shown in Figure 5(b4,b5) [40]. Compared with PLA, PLA/Biochar20, and PLA/Biochar30, the MH-containing formulations formed a more compact and shape-retained residue after combustion, suggesting that the MgO-containing residue reinforced the biochar-derived carbonaceous structure and improved the continuity of the protective barrier during burning [41].

The highest LOI value of 26.2% is achieved by PLA/Biochar20/MH20, which also shows the highest residue yield of 28.86% from the TGA results. This result suggests that increasing MH content enhances flame retardancy more effectively than increasing biochar alone. The strong improvement in LOI is consistent with the higher TGA char residue, indicating that the combined biochar/MH system improves flame resistance mainly through smoke suppression, heat absorption, gas dilution, and formation of stable char/inorganic residue (Figure 5(b6)). This multi-mechanism flame-retardant behavior is consistent with recent work emphasizing the importance of combining sustainable additives and design strategies to improve the fire performance of biodegradable polymer composites [42]. Overall, the results demonstrate that biochar provides a char-forming framework, while MH further strengthens the flame-retardant effect through endothermic dehydration and MgO formation. It is worth noting that an LOI of 21% is a practical threshold because it corresponds to the oxygen concentration in air [43]. Materials with LOI values above 21% are considered self-extinguished and can show better resistance to sustained burning under ambient conditions [44]. Therefore, the MH-containing composites, with LOI values of 22.5–26.2%, exceed this threshold and can be considered to exhibit good flame resistance.

UL-94 vertical burning tests were also performed to complement the LOI analysis, and the results are summarized in Table 2. Neat PLA, PLA/Biochar20, and PLA/Biochar30 composites continued to burn and exhibited melt dripping; therefore, they could not be assigned a UL-94 rating. By contrast, the composites containing MH were able to self-extinguish and showed no dripping during the test, demonstrating the important role of MH in limiting vertical flame spread. PLA/Biochar20/MH10 and PLA/Biochar30/MH10 reached V-2 ratings, whereas increasing the MH content by an additional 10 wt.% MH in the PLA/Biochar20/MH10 formulation further improved the rating to V-1. This behavior agrees well with the LOI results, confirming that the combined use of biochar and MH enhances flame suppression and promotes self-extinguishing performance.

It should be noted that the proposed barrier effect is inferred from the increased TGA residue, improved LOI and UL-94 performance, and the more compact post-combustion residue morphology; further char characterization, such as SEM, Raman spectroscopy, or XPS, would be needed to directly confirm the detailed structure and composition of the protective layer.

### 3.5. Mechanical Properties

**Figure 6:**
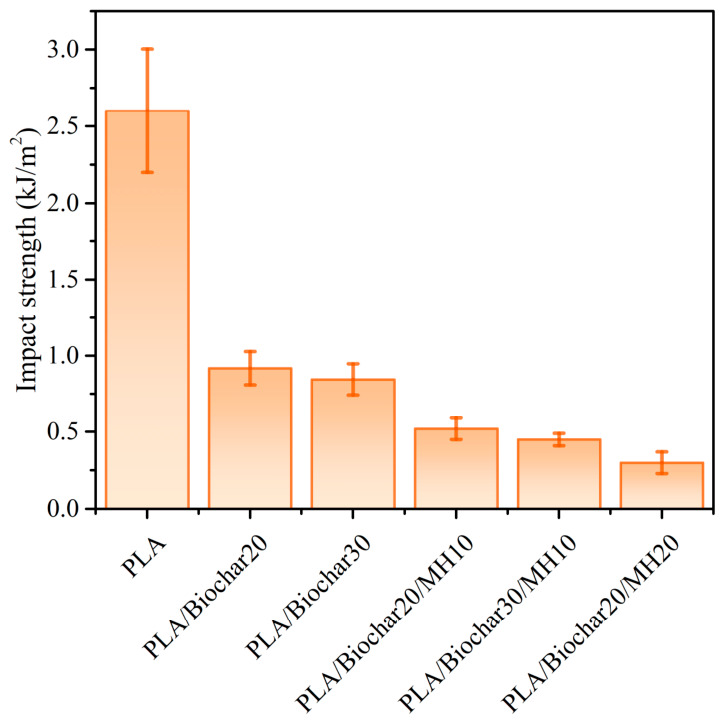
Impact strength of PLA composites. Error bars represent standard deviations based on five specimens.

The impact strength of PLA and its composites is shown in Figure 6. Neat PLA exhibits an impact strength of 2.60 kJ/m^2^, reflecting the brittle nature of PLA under sudden loading. After the incorporation of biochar, the impact strength decreases to 0.92 kJ/m^2^ for PLA/Biochar20 and 0.85 kJ/m^2^ for PLA/Biochar30. This reduction is mainly attributed to the rigid carbonaceous nature of biochar, which restricts local deformation of the PLA matrix and introduces stress-concentration sites under impact loading. Similar trends have been reported for PLA/biochar composites, where mechanical properties depend strongly on filler loading, dispersion, and interfacial adhesion [34].

With the further addition of MH, the impact strength decreases to 0.52 kJ/m^2^ for PLA/Biochar20/MH10, 0.45 kJ/m^2^ for PLA/Biochar30/MH10, and 0.31 kJ/m^2^ for PLA/Biochar20/MH20. The decrease can be mainly attributed to the increased total filler loading and the presence of rigid inorganic MH particles, which increase composite heterogeneity and reduce the ability of the PLA matrix to dissipate impact energy. At higher filler contents, particle agglomeration, weak interfacial regions, void formation, and stress concentration become more pronounced, accelerating crack initiation and propagation during impact [45]. In addition, MH may slightly affect PLA during melt processing through moisture- or hydroxyl-assisted ester bond scission, which could further contribute to matrix embrittlement and reduced impact resistance. This interpretation is consistent with the SEM observations, where MH-containing composites show rougher fracture surfaces, exposed particles, and localized voids.

The pronounced decrease in impact strength indicates an important limitation of the current PLA/Biochar/MH composite system. Although biochar and MH improve flame resistance and residue formation, their rigid particle structures reduce the ability of the PLA matrix to deform and absorb impact energy. Similar decreases in toughness have been reported for PLA/biochar and other highly filled PLA composites, where impact performance is strongly affected by filler loading, dispersion quality, and interfacial adhesion [21,22,46]. Therefore, the present composites may be more suitable for applications where flame resistance and dimensional stability are prioritized over high impact resistance. To mitigate this limitation, future work could focus on improving filler–matrix compatibility through biochar surface modification, silane treatment, or other coupling agents, the use of compatibilizers, incorporation of toughening modifiers such as flexible biodegradable polymers, and optimization of filler loading and processing conditions to reduce agglomeration and void formation.

### 3.6. Water Absorption and Accelerated Heat Aging

**Figure 7:**
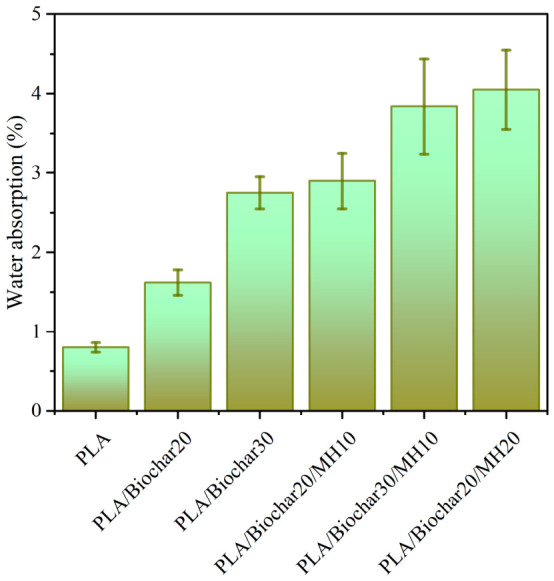
Water absorption percentage of PLA composites. Error bars represent standard deviations based on three specimens.

The water absorption behavior of PLA and its composites after 48 h of immersion is shown in Figure 7. Although neat PLA exhibits the lowest water uptake (0.80%) among the studied formulations, its absorption is still appreciable compared with nonpolar polyolefins [47] since PLA contains polar ester groups that can interact with water molecules. Water diffusion is also more likely to occur through the amorphous regions of PLA [48]. After biochar incorporation, water absorption increased to 1.62% and 2.75% for PLA/Biochar20 and PLA/Biochar30, respectively, likely due to the porous structure of biochar and the presence of oxygen-containing surface groups that can interact with water molecules. The further addition of MH results in a continued increase in water uptake, up to 4.05% for PLA/Biochar20/MH20, which may be attributed to the hydrophilic nature of MH, higher total filler loading, and increased interfacial regions between the PLA matrix and filler particles. These interfaces, together with possible micro-voids or localized defects, can provide additional pathways for water diffusion.

Despite the increase in water uptake after filler incorporation, the values obtained in this study remain reasonable compared with many filler-reinforced PLA composites. For example, PLA/wood fiber composites have been reported to show water absorption up to 11% with increasing fiber content, while PLA–carbon composites exhibited moisture uptake as high as 21% after 30 days of exposure [49]. In another study, natural fiber addition increased the water absorption of PLA from 0.25% for neat PLA to 2.70% after only 24 h, even at a relatively low fiber loading of 4.2 wt.%, due to the porous structure and hydrophilic sites introduced by the fibers [50]. Therefore, although biochar and MH slightly increase the water absorption of PLA in the present work, the relatively low uptake after 48 h indicates that the PLA matrix still provides an effective barrier against short-term water penetration.

**Figure 8:**
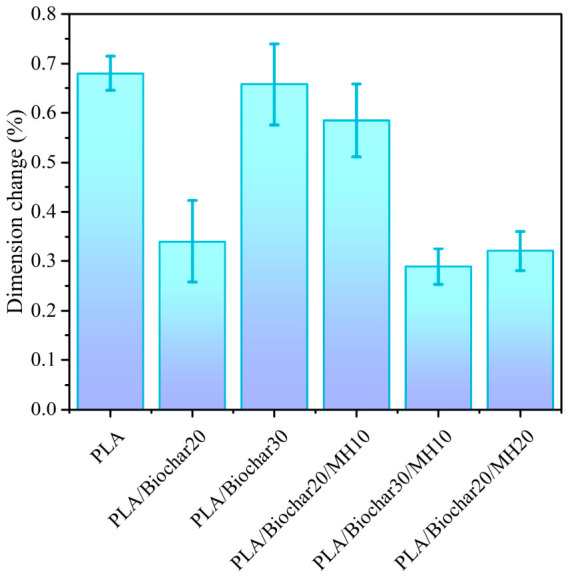
Accelerated heat aging dimension change in PLA composites. Error bars represent standard deviations based on three specimens.

The dimensional stability of PLA and its composites was assessed after accelerated aging at 60 °C. After 48 h of exposure, no obvious macroscopic deformation was observed, and the specimens generally maintained their original appearance as well as their length and width. However, thickness measurements revealed that thermal aging caused slight but measurable expansion in all samples. As shown in Figure 8, all samples exhibited a slight increase in thickness, with dimensional changes ranging from 0.29% to 0.68% after 48 h. No consistent composition-dependent trend was observed among the samples, which may be related to differences in thermal expansion behavior, filler distribution, and possible moisture interactions during aging. It should be noted that the aging experiment was conducted without humidity control, which is an important limitation because moisture uptake may contribute to dimensional changes in PLA-based composites, particularly in the presence of hydrophilic biochar and MH additives. Nevertheless, the observed dimensional changes were minor, remaining below 1%, and are therefore unlikely to cause severe deformation or significant practical performance issues. These results indicate that PLA and its composites maintained reasonable dimensional stability under elevated-temperature aging, although further optimization may still be beneficial for long-term use.

## 4. Conclusions

In this study, sustainable PLA composites were successfully prepared by melt compounding and compression molding. The use of biodegradable PLA as the polymer matrix, together with raw wood-derived biochar as a degradable carbon-based filler, provides a promising approach for developing more sustainable and value-added polymer composites. SEM and FTIR results confirmed the successful incorporation of biochar and MH into the PLA matrix, while TGA showed that the composites produced much higher final residues than neat PLA due to biochar-derived char formation and MgO residue from MH. The flame-retardant performance was significantly improved by the combined biochar/MH system. The best formulation, PLA/Biochar20/MH20, achieved the highest LOI of 26.2%, indicating effective condensed-phase protection through char formation, heat absorption, gas dilution, and MgO-assisted barrier formation. Although impact strength decreased with increasing filler loading and water absorption increased slightly, the composites maintained reasonable dimensional stability after accelerated aging, with thickness changes below 1%. Overall, these results demonstrate that incorporating biochar into biodegradable PLA, together with MH, is an effective halogen-free strategy for improving flame resistance based on LOI and UL-94 results and residue formation while enhancing the sustainable value of PLA-based composites.

## Figures and Tables

**Table 1:** Composition of PLA/Bio/MH composites.

Sample	PLA (wt.%)	Biochar (wt.%)	MH (wt.%)
Neat PLA	100	0	0
PLA/Biochar20	80	20	0
PLA/Biochar30	70	30	0
PLA/Biochar20/MH10	70	20	10
PLA/Biochar30/MH10	60	30	10
PLA/Biochar20/MH20	60	20	20

**Table 2:** UL-94 vertical flammability results of PLA/Bio/MH composites.

Sample	Self-Extinguished	Dripping	UL-94 Rating
Neat PLA	No	Yes	No rating
PLA/Biochar20	No	Yes	No rating
PLA/Biochar30	No	Yes	No rating
PLA/Biochar20/MH10	Yes	No	V-2
PLA/Biochar30/MH10	Yes	No	V-2
PLA/Biochar20/MH20	Yes	No	V-1

## Data Availability

The original contributions presented in this study are included in the article. Further inquiries can be directed to the corresponding authors.
